# Supplementary material for: Bioinformatics and Connectivity Map Analysis Suggest Viral Infection as a Critical Causative Factor of Hashimoto’s Thyroiditis
Source: Int J Mol Sci. 2023 Jan 6;24(2):1157. doi: 10.3390/ijms24021157 (PMC9865288; doi:10.3390/ijms24021157)
Supplement: Supplementary file 1 [file ijms-24-01157-s001.zip › Supplementary data.pdf]

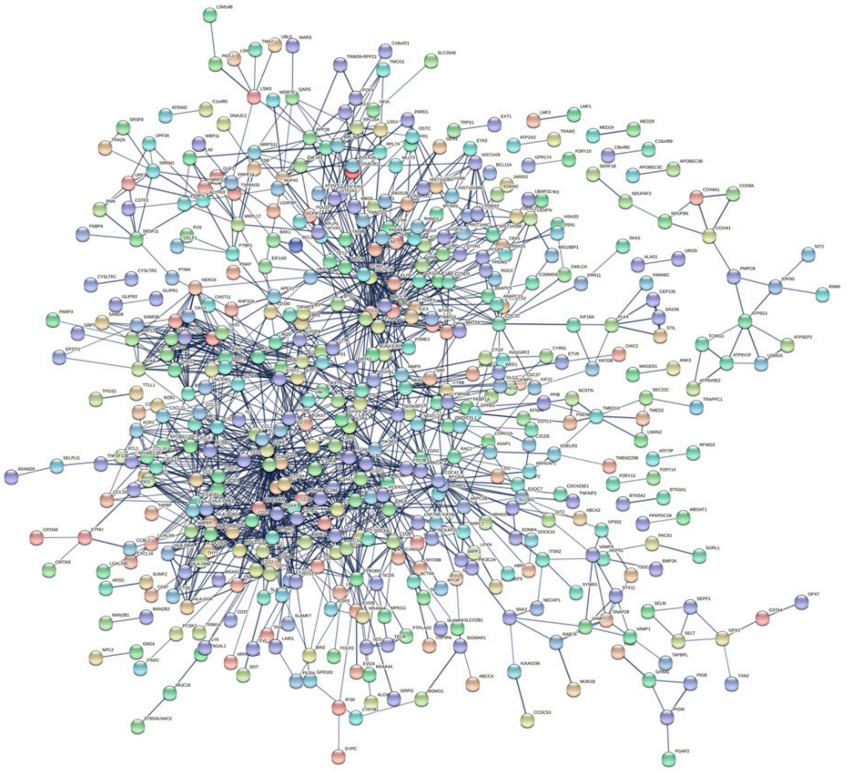

Figure S1 PPI network of upregulated DEGs in the HT group obtained from thyroid tissue microarray data.

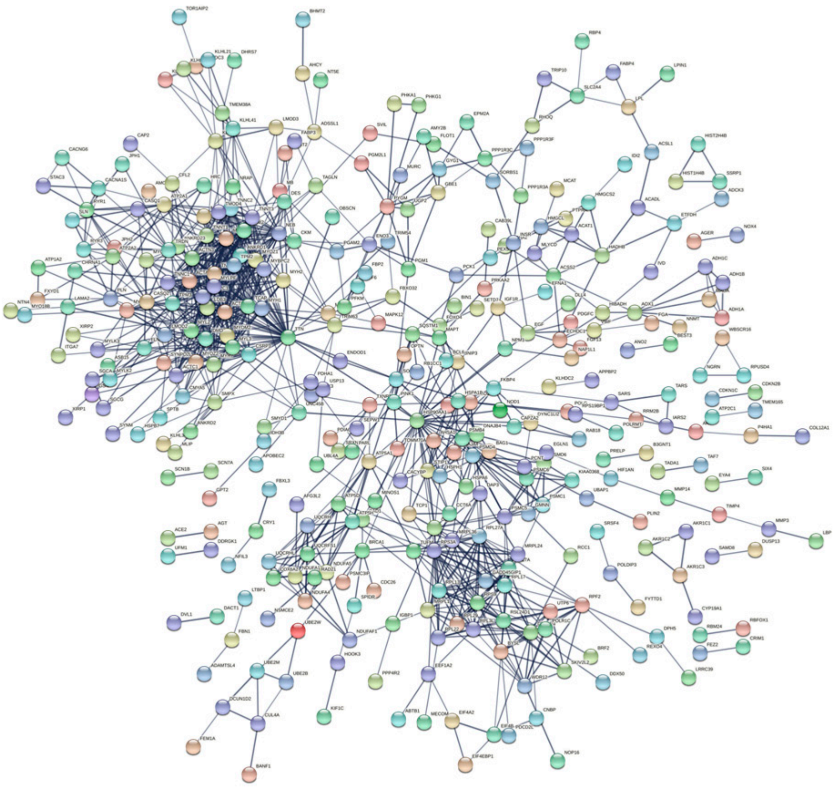

Figure S2 Total PPI network of downregulated DEGs in the HT group obtained from thyroid tissue microarray data.

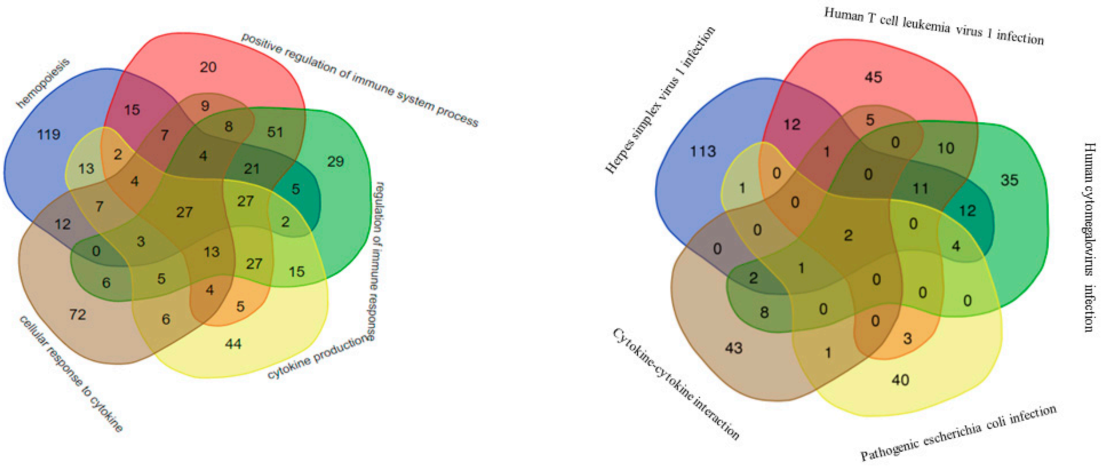

Figure S3 Venn diagram showing gene distribution for the top five significant (A) BP terms and (B) KEGG pathways of the HT group. Total gene lists are provided as a supplementary data file.
